# Seed-Specific Overexpression of *SPL12* and *IPA1* Improves Seed Dormancy and Grain Size in Rice

**DOI:** 10.3389/fpls.2020.532771

**Published:** 2020-09-03

**Authors:** Miaomiao Qin, Yan Zhang, Yanmei Yang, Chunbo Miao, Shenkui Liu

**Affiliations:** State Key Laboratory of Subtropical Silviculture, School of Forestry and Biotechnology, Zhejiang A&F University, Hangzhou, China

**Keywords:** seed dormancy, seed germination, *SPL12*, *IPA1*, gibberellin pathway

## Abstract

Pre-harvest sprouting (PHS) often results in reduced grain yield and quality and is a major problem in cereal production. Improved seed dormancy would inhibit PHS. Here we show that seed-specific overexpression of two *SQUAMOSA-PROMOTER BINDING PROTEIN-LIKE* (*SPL*) genes *SPL12* and *IPA1* enhances seed dormancy and inhibits PHS without noticeable effects on shoot architecture in rice. In addition, seed-specific overexpression of *IPA1* also increases grain size and thus improves grain productivity. Furthermore, our results suggest that *SPL12* enhances the seed dormancy through directly regulating many genes in the gibberellin (GA) pathway. This research provides an efficient method to suppress PHS and will facilitate breeding elite crop varieties.

## Introduction

In agriculture, pre-harvest sprouting (PHS) often results in severe grain yield loss, as well as reduced grain quality and germinability (Li et al., 2004; Chono et al., 2006), and thus imposes a major threat to agriculture and food security. In crops, seed dormancy is regarded as an important agronomic trait due to its key roles in controlling PHS and seed longevity (Gubler et al., 2005; Kottearachchi et al., 2006; Wu et al., 2016). Improved seed dormancy would inhibit PHS and increase seed longevity. Therefore, controlling seed dormancy is a very important goal in agriculture worldwide.

Seed dormancy is first acquired during seed mature stage, and the phytohormone abscisic acid (ABA) plays a fundamental role in establishing this primary seed dormancy. ABA positively regulates seed dormancy, and as seeds mature, ABA level gradually increases to establish seed dormancy (Kang et al., 2015). Besides ABA, another key factor determining seed dormancy is the phytohormone gibberellins (GAs) (Shu et al., 2016; Tuan et al., 2018). GAs promote the transition from dormancy to germination, and antagonize the effects of ABA on seed dormancy. Therefore, the balance between the effects of ABA and GAs is crucial in determining the status of seed dormancy. Many other factors, such as the phytohormone auxins, temperature, light, and air humidity, also have effects on seed dormancy, and most of these factors affect seed dormancy through the ABA and/or GA pathways (Finch-Savage and Footitt, 2017). Although major progress in studying seed dormancy have been made, the detailed molecular mechanisms underlying seed dormancy are largely unknown, and crop breeders often lack effective methods to control seed dormancy.

The conserved microRNA miR156, together with its targets *SQUAMOSA-PROMOTER BINDING PROTEIN-LIKE* (*SPL*) transcription factor genes, plays important roles in many aspects of plant development (Chen et al., 2010; Preston and Hileman, 2013). In crops, the miR156/SPL module regulates grain yield through modulating plant shoot architecture and grain size (Wang, 2014; Wang and Wang, 2015). In rice, the miR156 target gene *Ideal Plant Architecture 1* (*IPA1*) encodes SPL14 and was recently considered as a “green revolution” gene for improving grain yield (Lu et al., 2013; Wang and Wang, 2017). The *IPA1* quantitative trait locus (QTL) allele *ipa1-1d* relieves its suppression by miR156 and thus confers an ideal plant architecture with fewer tillers, stronger culms, and more grains per panicle (Jiao et al., 2010). Recently, miR156 was reported to negatively regulate seed dormancy, and *IPA1* mediated part of the effects of miR156 on seed dormancy by directly regulating many genes in the GA pathway (Miao et al., 2019). However, the roles of other *SPL* genes such as *SPL12* in seed dormancy are still unknown.

Here, to explore the roles of *SPL* genes in seed development, we examined the effects of *SPL12* and *IPA1* overexpression (*P_Ole18_:SPL12* and *P_Ole18_:IPA1*) on seed dormancy and grain size. Our results showed that overexpression of *IPA1* and *SPL12* driven by the seed-specific *Ole18* promoter (Wu et al., 1998) enhanced seed dormancy and inhibited PHS without obvious effects on plant shoot architecture. In addition, *IPA1* seed-specific overexpression also increased grain size and improved grain productivity. Furthermore, our results showed impaired GA pathway in *SPL12* overexpression lines and revealed *in vivo* associations of SPL12 with the promoters of many GA biosynthetic, deactivating and signaling genes, suggesting that *SPL12* positively regulates seed dormancy through directly regulating the genes in the GA pathway. Our results provide an effective method to inhibit PHS and will facilitate breeding elite crop varieties.

## Materials and Methods

### Vector Construction and Plant Materials

To construct *P_Ole18_:SPL12* and *P_Ole18_:IPA1* vectors, the *Ole18* promoter (a 1249-bp fragment upstream the start codon in the *Ole18* gene) (Wu et al., 1998) was first fused respectively with *SPL12* and *IPA1*-encoding sequences through fusion PCR technology, and then the *P_Ole18_:SPL12* and *P_Ole18_:IPA1* fragments were introduced into the intermediate vector pDONR207 using Gateway™ BP Clonase™ II Enzyme Mix (Invitrogen, Cat. no. 11789-020). After sequencing characterization, the *P_Ole18_:SPL12* and *P_Ole18_:IPA1* fragments were subcloned from the right pDONR207 (*P_Ole18_:SPL12*) and pDONR207 (*P_Ole18_:IPA1*) clones, respectively, into the pGWB4 vector using Gateway™ LR Clonase™ II Enzyme Mix ((Invitrogen, Cat. no. 11791-020). The resulting overexpression vectors were transformed into the *Japonica* rice variety Zhonghua 11 (ZH11) through *Agrobacterium*-mediated method.

To construct *P_35S_:SPL12-FLAG* overexpression vector, the *SPL12*-encoding sequence was cloned into intermediate vector pDONR207 using Gateway™ BP Clonase™ II Enzyme Mix (Invitrogen, Cat. no. 11789-020), and then the *SPL12*-encoding sequence was subcloned from the pDONR207 (*P_35S_:SPL12-FLAG*) clone into the pGWB11 vector using Gateway™ LR Clonase™ II Enzyme Mix ((Invitrogen, Cat. no. 11791-020). The resulting *P_35S_:SPL12-FLAG* overexpression vector was transformed into the *Japonica* rice variety Xiushui 134 (XS134) through *Agrobacterium*-mediated method.

The transgenic plants were grown in the paddy field. The seeds from the transgenic plants were sowed in Hangzhou (China) in early June.

### RT-qPCR

Total RNA was extracted using RNAprep Pure Plant Kit (TIANGEN, Cat. No. DP432, China). Reverse transcription was performed using PrimeScriptTM RT reagent Kit (Takara, Cat. No. RR047A, Japan). Real-time PCR assays were performed using SYBR^®^ Premix Ex Taq™ II (Takara, Cat. No. RR820A, Japan), and the Bio-Rad CFX96 real-time PCR instrument was used in our study. The sequences of gene-specific primers for the target genes were listed in **Table S3**, and rice *Ubiquitin* gene (LOC_Os05g06770) was used as an internal reference gene, and qPCR reactions were performed in three biological replicates.

### ChIP-qPCR

ChIP assays were performed using ab117137-ChIP Kit-Plants (abcam, Lot. GR323561-19, England). Firstly, we harvested 1 g seedling shoots from *P_35S_:SPL12-FLAG* transgenic plants when the seedlings were grown to about 15 cm in the paddy field. Then the chromatin complexes were isolated, sonicated and incubated with anti-FLAG antibody named M185-3LL (Japan). Precipitated chromatin DNA was analyzed by quantitative PCR using SYBR^®^ Premix Ex Taq™ II (Takara, Cat. No. RR820A, Japan). Primer sequences for the real-time PCR were listed in **Table S4**, and qPCR reactions were performed in three biological replicates.

### Seed Dormancy and Germination

Undehusked fresh seeds without drying treatment were first soaked in water for two days (16 h light/8 h dark cycles at 30°C), and then continued to germinate at 30°C. The germination rate was counted every two days.

### PHS Investigation

PHS was investigated in the paddy field of Hangzhou at the normal harvest time. In every line, all of the seeds from a plant were investigated in the assays, and the sprouting data of every line were obtained from at least three plants. The PHS investigations were finished in 1 day.

### Plot Grain Yield Test

Seedlings of the wild type, *P_Ole18_*:*SPL12* and *P_Ole18_*:*IPA1* were grown in Hangzhou paddy field under natural conditions. The area per plot was 90 cm×60 cm, and 24 plants were cultivated in each plot with planting density of 15 cm×15 cm.

### Phytohormone Measurement

Plant materials were ground into powder in liquid nitrogen, and extracted with methanol/water (8/2) at 4°C. The extract was centrifuged at 12,000 g under 4°C for 15 min. The supernatant was collected and evaporated to dryness under nitrogen gas stream, and then reconstituted in methanol/water (3/7). The solution was centrifuged and the supernatant was collected for LC-MS analysis. The LC-MS analysis was conducted with the API6500 Q TRAP LC/MS/MS system, equipped with an ESI Turbo Ion-Spray interface, operating in a positive ion mode and controlled by Analyst 1.6 software (AB Sciex).

## Results

### Seed-Specific Overexpression of *SPL12* and *IPA1* Enhanced Seed Dormancy

**Figure 1:**
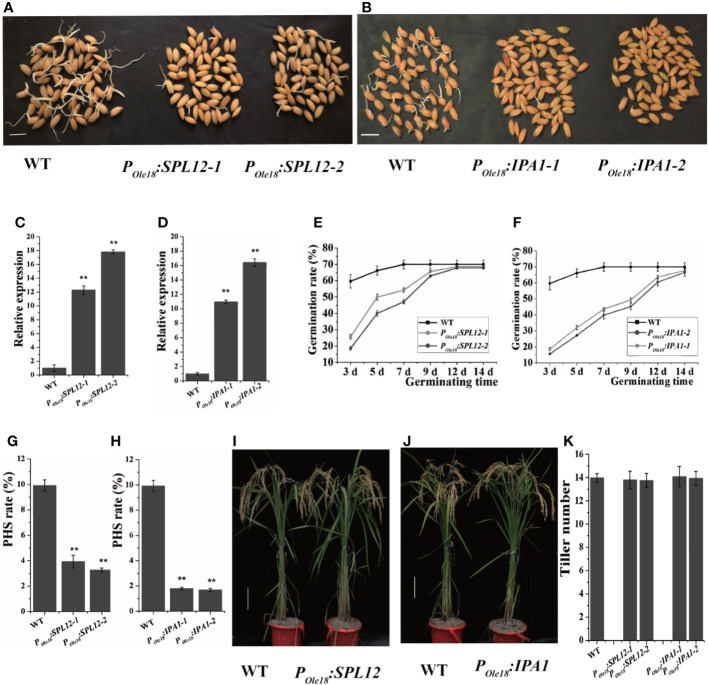
Seed-specific overexpression of *SPL12* and *IPA1* enhanced seed dormancy. **(A)** Germination of fresh seeds of *P_Ole18_:SPL12* and wild-type lines. Scale bar, 1 cm. **(B)** Germination of fresh seeds of *P_Ole18_:IPA1* and wild-type lines. Scale bar, 1 cm. **(C)** Relative expression levels of *SPL12* in the fresh seed embryos of wild-type and *P_Ole18_:SPL12* lines. **(D)** Relative expression levels of *IPA1* in the fresh seed embryos of wild-type and *P_Ole18_:IPA1* lines. **(E)** Germination rates of fresh seeds of wild-type and *P_Ole18_:SPL12* lines. **(F)** Germination rates of fresh seeds of wild-type and *P_Ole18_:IPA1* lines. **(G)** PHS rates of wild-type and *P_Ole18_:SPL12* lines. **(H)** PHS rates of wild-type and *P_Ole18_:IPA1* lines. **(I)** Wild-type and *P_Ole18_:SPL12* plants at the mature stage. Scale bar, 10 cm. **(J)** Wild-type and *P_Ole18_:IPA1* plants at the mature stage. Scale bar, 10 cm. **(K)** Tiller numbers of wild-type, *P_Ole18_*:*SPL12* and *P_Ole18_*:*IPA1* plants. The double asterisks (**) represent significant difference calculated by the Student’s *t*-test at *P* < 0.01. Values are means ± SE (n = 3).

Disrupting *MIR156* genes was recently reported to enhance seed dormancy in rice (Miao et al., 2019), and transcriptomic data published previously showed that among the miR156 target genes, *IPA1* and *SPL12* were expressed most highly in the embryos of fresh wet seeds (freshly harvested seeds without drying treatment, and termed fresh seeds hereinafter), suggesting that both *IPA1* and *SPL12* were involved in seed dormancy controlling. Thus, we overexpressed *IPA1* and *SPL12* using the rice seed-specific *Ole18* promoter (Wu et al., 1998), and at least twenty-five independent lines of each vector were obtained. Germination analyses using fresh seeds revealed that seed-specific overexpression lines of *SPL12* and *IPA1* showed obviously slower germination than the wild type (**Figures 1A–F**). These results indicate that overexpression of *SPL12* and *IPA1* enhances seed dormancy. In addition, we also investigated the PHS rates of *P_Ole18_:SPL12* and *P_Ole18_:IPA1* overexpression lines and the wild type in the paddy field of Hangzhou, and found that overexpression of *SPL12* and *IPA1* significantly suppressed PHS (**Figures 1G, H**).

In crops, the miR156/SPL module plays key roles in modulating plant shoot architecture. Thus, we examined the plant shoot architectures of the *SPL12* and *IPA1* overexpression lines, and observed no obvious differences between the overexpression lines and the wild type (**Figures 1I–K**). In fact, besides seed dormancy and grain size, we observed no obvious differences between the overexpression lines and the wild type.

### Overexpression of *IPA1* but Not *SPL12* Increased Grain Size

**Figure 2:**
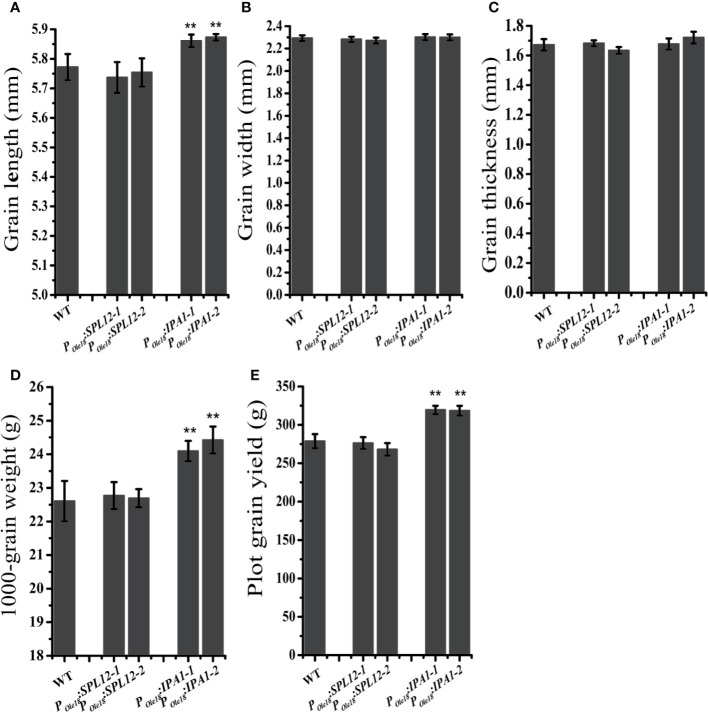
Overexpression of *IPA1* but not *SPL12* increased grain size. **(A)** Grain lengths of wild-type, *P_Ole18_*:*SPL12* and *P_Ole18_*:*IPA1* plants. **(B)** Grain width of wild-type, *P_Ole18_*:*SPL12* and *P_Ole18_*:*IPA1* plants. **(C)** Grain thickness of wild-type, *P_Ole18_*:*SPL12* and *P_Ole18_*:*IPA1* plants. **(D)** 1,000-grain weights of wild-type, *P_Ole18_*:*SPL12* and *P_Ole18_*:*IPA1* plants. **(E)** Plot grain yield of wild-type, *P_Ole18_*:*SPL12* and *P_Ole18_*:*IPA1* plants. The double asterisks (**) represent significant difference calculated by the Student’s *t*-test at *P* < 0.01. Values are means ± SE (n = 3).

In crops, the miR156/SPL module has important roles in modulating the grain size and shape. Thus, we compared the grain sizes and shapes of the overexpression lines to the wild type. We did not observe obvious differences in grain size and shape between the *SPL12* overexpression lines and the wild type (**Figures 2A–D**), suggesting that *SPL12* plays negligible roles in modulating grain size and shape. Unlike the situation in *SPL12* overexpression lines, *IPA1* overexpression significantly increased grain lengths and 1000-grain weights (**Figures 2A–D**). Consistently, plot grain yield test revealed that *IPA1* overexpression slightly increased the grain yield (**Figure 2E**).

### Overexpression of *SPL12* Enhanced Seed Dormancy Through the GA Pathway

**Figure 3:**
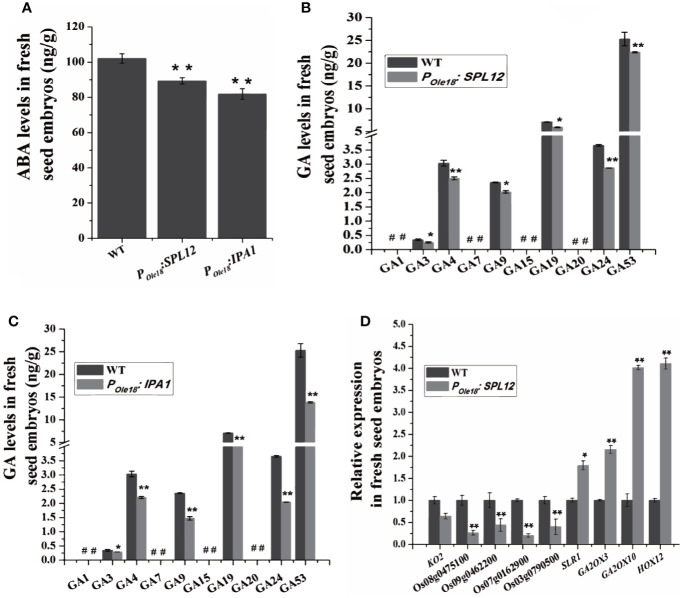
Overexpression of *SPL12* enhanced seed dormancy through GA pathway. **(A)** ABA levels in wild-type, *P_Ole18_*:*SPL12*, and *P_Ole18_*:*IPA1* fresh seed embryos. **(B)** GA levels in wild-type and *P_Ole18_*:*SPL12* fresh seed embryos. #, undetectable GAs. **(C)** GA levels in wild-type and *P_Ole18_*:*IPA1* fresh seed embryos. #, undetectable GAs. **(D)** Relative expression levels of GA biosynthetic, signaling, and deactivating DEGs in fresh seed embryos. Three biological replications were performed in each assay. The double asterisks (**) represent significant difference calculated by the Student’s *t*-test at *P* < 0.01, and single asterisk (*) means *P* < 0.05. Values are means ± SE (n = 3).

To explore how overexpression of *SPL12* enhanced seed dormancy, we analyzed the transcriptomes of the fresh seed embryos from a *SPL12* overexpression line (designated *SPL12-2*) and the wild type, and the result did not suggest enhanced ABA pathway in the *SPL12* overexpression line compared to the wild type. Phytohormone measuring revealed that compared to the wild type, the fresh seed embryos of *SPL12* and *IPA1* overexpression lines contained decreased ABA levels (**Figure 3A**). These results suggest that overexpression of *SPL12* and *IPA1* does not enhance seed dormancy through the ABA pathway.

Then, we measured GA levels in the fresh seed embryos. The results showed that the levels of two bioactive GAs, GA_3_, and GA_4_, were lower in *SPL12* and *IPAI* overexpression lines than in the wild type (**Figures 3B, C**). We could not find other bioactive GAs (GA_1_ and GA_7_) in the fresh seed embryos of both overexpression and wild-type lines.

Consistent with the recent report that the GA pathway was impaired by the *MIR156* gene disruption, our transcriptome analyses showed that *SPL12* overexpression down-regulated some key GA biosynthetic and putative GA receptor genes but up-regulated some GA deactivating genes (**Table S1** and **Figure S1**), suggesting impaired GA pathway in the fresh seed embryos of *SPL12* overexpression lines. In the *SPL12-2* overexpression line compared to the wild type, a total of 3632 differentially expressed genes (DEGs), including 2239 up-regulated and 1393 down-regulated genes, were identified (ratio ≥ 2 or ≤ 0.5, and false discovery rate (FDR) < 0.05) (**Table S2**). Among the DEGs identified in the *SPL12-2* overexpression line compared to the wild type, three important GA deactivating genes (*GA2ox3*, *GA2ox10*, and *HOX12*) were up-regulated, whereas four putative GA receptor genes (Os07g0162900, Os08g0475100, Os09g0462200, and Os03g0790500) were obviously down-regulated by the overexpression (**Table S1**). When lowering the threshold for DEGs to ratio ≥ 1.5 or ≤ 0.75 (FDR < 0.05), we found that a key GA biosynthetic gene *KO2* was also down-regulated, whereas *SLR1*, one negative regulator of GA signaling, was up-regulated (**Table S1**). These results were validated by RT-qPCR (**Figure 3D** and **Table S3**). These results suggest that *SPL12* enhances seed dormancy through the GA pathway.

### Associations Between SPL12 and the Promoters of GA Biosynthetic, Signaling and Deactivating Genes

**Figure 4:**
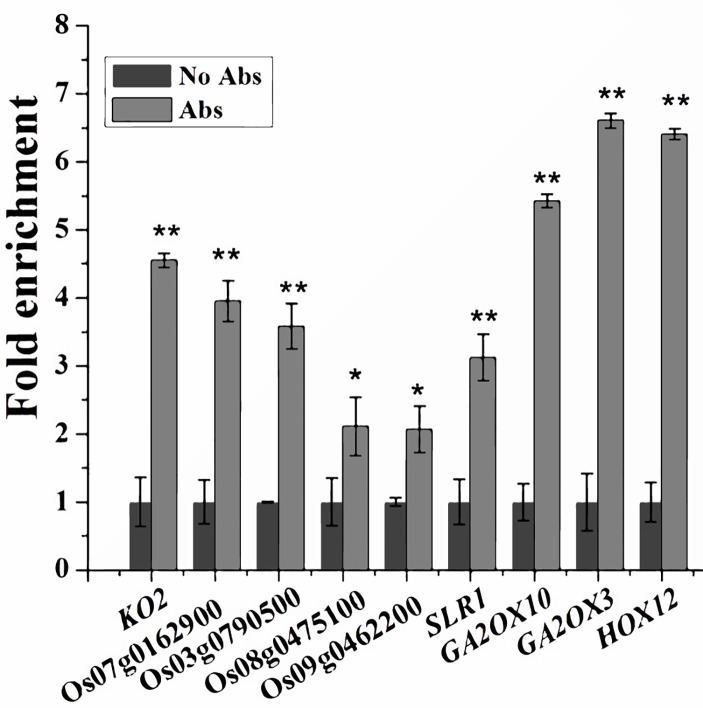
Associations between SPL12 and the promoters of GA biosynthetic, signaling, and deactivating genes. Primers for the ChIP-qPCR were listed in Values are means ± SE (n = 3). The double asterisks (**) represent significant difference calculated by the Student’s *t*-test at *P* < 0.01, and single asterisk (*) means *P* < 0.05. The fold enrichment was normalized against the promoter of rice *Ubiquitin*.

Since SPL12 is a transcription factor, it is possible that SPL12 directly regulates GA-related genes. SPL family proteins were reported to directly bind GTAC motifs to regulate gene expression (Birkenbihl et al., 2005; Lu et al., 2013). Through sequence analyses of the 1000-bp promoter regions, we found that most of the GA-related DEGs identified above contained multiple GTAC motifs. Thus, we conducted ChIP-qPCR assays to explore the possible interactions between SPL12 and the promoters of GA-related DEGs. The results revealed direct interactions between SPL12 and 9 GA-related genes (**Figure 4**). The 9 genes included one GA biosynthetic gene *KO2*, four putative GA receptor genes (Os07g0162900, Os03g0790500, Os09g0462200, and Os08g0475100), three GA deactivating genes (*GA2ox3*, *GA2ox10*, and *HOX12*), and *SLR1*. These results suggest that SPL12 controls the GA pathway through directly regulating the GA biosynthetic, signaling, and deactivating genes.

## Discussion

In crops, PHS often results in severe grain yield loss and reduced grain quality. Improved seed dormancy would inhibit PHS and increase seed longevity (Gubler et al., 2005; Kottearachchi et al., 2006; Wu et al., 2016). The balance between the effects of ABA and GAs is crucial in determining the status of seed dormancy (Shu et al., 2016; Tuan et al., 2018). In rice, miR156 was reported to negatively regulate seed dormancy, and *IPA1* mediated part of the effects of miR156 on seed dormancy (Miao et al., 2019). Among the *SPL* family genes, *SPL13*, *IPA1*, and *SPL16* were reported to positively regulate seed size (Wang et al., 2012; Wang et al., 2015; Si et al., 2016). However, the roles of *SPL12* in seed dormancy and seed size are still unknown.

In this study, we found that seed-specific overexpression of *SPL12* and *IPA1* improved seed dormancy with negligible effects in rice plant architecture (**Figures 1I–K**). *SPL12-2* overexpression line enhanced seed dormancy more intensely than *SPL12-1* overexpression line, which was consistent with higher *SPL12* expression level in *SPL12-2* overexpression line than that in *SPL12-1* overexpression line. Seed-specific overexpression of *IPA1* significantly increased seed lengths, 1,000-grain weights and the grain yield, but seed-specific overexpression *SPL12* had negligible effects in seed size. *IPA1* but not *SPL12* increased grain size, and we inferred that it could be due to the different downstream genes regulated by SPL12 and IPA1.

Phytohormone measuring revealed that compared to the wild type, the fresh seed embryos of *P_Ole18_:SPL12-2* and *P_Ole18_:IPA1-2* overexpression lines contained slightly decreased ABA levels, and consistently, the transcriptome analyses did not reveal enhanced ABA pathway in *P_Ole18_:SPL12* fresh seed embryos compared to the wild type, suggesting that overexpression of *SPL12* and *IPA1* did not enhance seed dormancy through the ABA pathway. The phytohormone measuring also revealed decreased bioactive GA (GA_3_ and GA_4_) levels, suggesting that *SPL12* may enhance seed dormancy through the GA pathway. Previous study showed that *IPA1* mediated the effects of miR156 on seed dormancy through regulating multiple genes in the GA pathway (Miao et al., 2019). Considering the similarity between IPA1 and SPL12, we inferred that *SPL12* also enhanced seed dormancy through the GA pathway.

Our transcriptomic analyses supported this speculation. The transcriptomic analyses in wild-type and *P_Ole_ : SPL12* fresh seed embryos showed that a key GA biosynthetic gene *KO2* and four putative GA receptor genes (Os07g0162900, Os08g0475100, Os09g0462200, and Os03g0790500) were obviously down-regulated by *SPL12* overexpression, whereas a negative regulator of GA signaling *SLR1* and three important GA deactivating genes (*GA2ox3*, *GA2ox10*, and *HOX12*) were up-regulated by *SPL12* overexpression, suggesting impaired GA pathway in the fresh seed embryos of *P_Ole18_:SPL12* overexpression lines. In addition, ChIP-qPCR assays demonstrated association of SPL12 with the promoters of a GA biosynthetic genes, four putative GA receptor genes, three deactivating genes and one negative regulator of GA signaling gene *SLR1*, suggesting that *SPL12* enhanced seed dormancy through directly regulating multiple genes in the GA pathway.

In general, our study supports that seed-specific overexpression of *SPL12* and *IPA1* enhances seed dormancy through directly regulating multiple genes in the GA pathway. *IPA1* seed specific overexpression also increases grain size and improves grain productivity. This research provides an efficient method to suppress PHS and will facilitate breeding elite crop varieties.

## Data Availability Statement

All datasets generated for this study are included in the article/**Supplementary Material**.

## Author Contributions

CM and MQ designed the study and constructed all vectors. MQ and YZ performed the transgenic experiments, analyzed data and wrote the main manuscript. YY and MQ analyzed data. CM and SL oversaw the entire study.

## Funding

This work was supported by the Zhejiang Science and Technology Major Program on Agricultural New Variety Breeding (Grant No. 2016C02056-1), the National Natural Science Foundation of China for Young Scientists (Grant. no. 31800241) and the Program for Changjiang Scholars and Innovative Research Team in University of the Ministry of Education of China (No. IRT17R99).

## Conflict of Interest

The authors declare that the research was conducted in the absence of any commercial or financial relationships that could be construed as a potential conflict of interest.
