# Supplementary material for: Seed-Specific Overexpression of SPL12 and IPA1 Improves Seed Dormancy and Grain Size in Rice
Source: Front Plant Sci. 2020 Sep 3;11:532771. doi: 10.3389/fpls.2020.532771 (PMC7509454; doi:10.3389/fpls.2020.532771)

Supplemental Fig. 1. Expression profiles of GA biosynthetic, signaling, and deactivating genes in wild-type and P_{ole18}:*SPL12* over-expression fresh seed embryos. The genes with decreased reads in *SPL12* over-expression line are highlighted in blue.

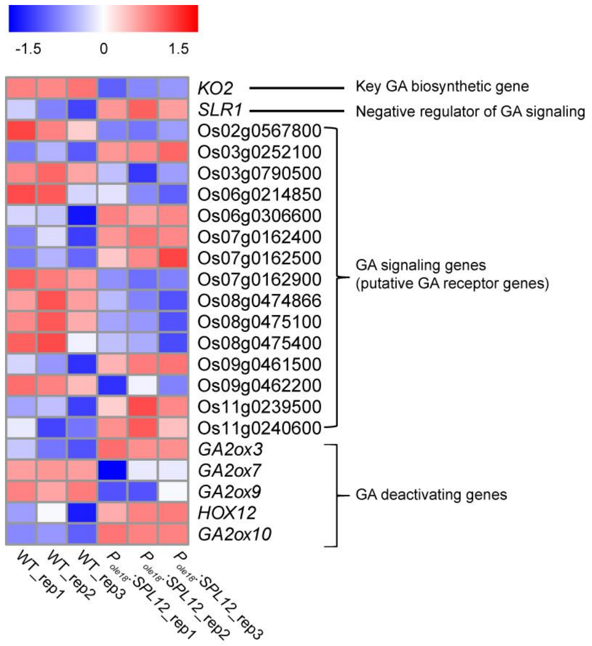

Supplement: Supplementary file 1 [file Image_1.pdf]
