# Supplementary material for: Seed-Specific Overexpression of SPL12 and IPA1 Improves Seed Dormancy and Grain Size in Rice
Source: Front Plant Sci. 2020 Sep 3;11:532771. doi: 10.3389/fpls.2020.532771 (PMC7509454; doi:10.3389/fpls.2020.532771)
Supplement: Supplementary file 2 [file Table_1.docx]

Supplemental Table 1. Expression profiles of GA-related genes in *SPL12* over-expression and wild-type fresh seed embryos

| Genes | Fold Change  (SPL12OE/WT) | log2^(SPL12OE/WT)^ | p_value | description |
| --- | --- | --- | --- | --- |
| *KO2* | 0.581011212 | -0.78336209 | 3.69764E-11 | key GA biosynthetic genes |
| Os08g0475100 | 0.291507791 | -1.778393652 | 1.66222E-07 | GA signaling genes |
| Os09g0462200 | 0.460152318 | -1.119816599 | 1.18835E-06 | GA signaling genes |
| Os07g0162900 | 0.139823858 | -2.838317551 | 6.79192E-19 | GA signaling genes |
| Os03g0790500 | 0.269059331 | -1.894003755 | 1.31417E-06 | GA signaling genes |
| *SLR1* | 1.727870756 | 0.788995308 | 3.10908E-18 | Negative regulators of GA signalig |
| *OsGA2ox3* | 2.046024716 | 1.032823573 | 0.030774937 | key GA deactivatig genes |
| *GA2OX10* | 4.310583168 | 2.107883061 | 1.59142E-14 | key GA deactivatig genes |
| *HOX12* | 3.535857218 | 1.822060018 | 0.000482133 | key GA deactivatig genes |
