# Supplementary material for: Seed-Specific Overexpression of SPL12 and IPA1 Improves Seed Dormancy and Grain Size in Rice
Source: Front Plant Sci. 2020 Sep 3;11:532771. doi: 10.3389/fpls.2020.532771 (PMC7509454; doi:10.3389/fpls.2020.532771)
Supplement: Supplementary file 4 [file Table_3.docx]

| Genes | Primers | Sequences (5’ to 3’) | length |
| --- | --- | --- | --- |
| *KO2* | KO2-6F | ACTCACCCGCTGTCACTGT | 199bp |
|  | KO2-6R | GGCGAATGCAAACTTGAT |  |
| Os08g0475100 | 5100-6F | CACTGCTACTTCCTCAACAACC | 167bp |
|  | 5100-6R | ATGGCTTTACTCGGGTGTGTAG |  |
| Os09g0462200 | 2200-6F | CACACGGCCTTCAACTTCGT | 144bp |
|  | 2200-6R | GAGGACAGCCTGCCACGAG |  |
| Os07g0162900 | 2900-6F | GCACCTGGCCCAACTGCCA | 126bp |
|  | 2900-6R | TCTCGGCGTCCTCGAACG |  |
| Os03g0790500 | 0500-6F | GTGCTCGTCTACTTCCACG | 120bp |
|  | 0500-6R | GTAGTCGGCGGAGAGGACGA |  |
| *SLR1* | SLR1-6F | TTGCAGCAGGTGGGTTGGAAG | 166bp |
|  | SLR1-6R | AGTTGACGGCGATCACCTCAG |  |
| *GA2ox3* | GA2ox3-6F | GTTCTTCAAGGTCGTCA | 196bp |
|  | GA2ox3-6R | GTCGAGGGCGAGGAGGAGGT |  |
| *GA2ox10* | GA2ox10-6F | CGTTGTGGCGGTGAGAG | 162bp |
|  | GA2ox10-6R | CTTCAACTCAGGGTGTGGTGG |  |
| *Hox12* | Hox12-6F | CTCGACGCCAAGCAGGT | 180bp |
|  | Hox12-6R | CTCCTCCTCTACATCC |  |
| *Ubiquitin* | Ubi-6F | ATCGACAACGTGAAGGC | 195bp |
| (LOC_Os05g06770) | Ubi-6R | CTTGGTGTACGTCTTCTTCT |  |

Supplemental Table 3. RT-qPCR primers of *SPL12* over-expression seed embryos
