# Supplementary material for: Seed-Specific Overexpression of SPL12 and IPA1 Improves Seed Dormancy and Grain Size in Rice
Source: Front Plant Sci. 2020 Sep 3;11:532771. doi: 10.3389/fpls.2020.532771 (PMC7509454; doi:10.3389/fpls.2020.532771)
Supplement: Supplementary file 5 [file Table_4.docx]

Supplemental Table 4. CHIP-qPCR primers

| Genes | Primers | Sequences (5’ to 3’) | length |
| --- | --- | --- | --- |
| *KO2* | KO2-2F | TCTAAGATTTACGGAGAAGTAC | 152bp |
|  | KO2-2R | CACAAAATGATATTTTCACAAG |  |
| *SLR1* | SLR1-2F | GTATGCATAGAGTGTGTACGTG | 176bp |
|  | SLR1-2R | GCCAATGCATCTTACAAAACTG |  |
| Os07g0162900 | 2900-1F | CTCGCCGTGCGGTTCGG | 161bp |
|  | 2900-1R | CGGTCGTTGAGGTCGCGGT |  |
| Os03g0790500 | 0500-2F | GTCGCCCTCCGCCTCGG | 117bp |
|  | 0500-2R | GTACTCCGCCTCCGACCT |  |
| Os08g0475100 | 5100-1F | GGGTCTCTTAAATGACCAGTG | 230bp |
|  | 5100-1R | TTGCTGCCTACGTACAACGAC |  |
| Os09g0462200 | 2200-1F | GAACGTGGTGAAGATGTGGA | 153bp |
|  | 2200-1R | TCGCGGATCACATCCTTCTC |  |
| *GA2OX10* | GA2OX10-1F | GCTCGTTCCGCGATATCT | 142bp |
|  | GA2OX10-1R | GAGTCACTCTCCTCGTGT |  |
| *HOX12* | HOX12-2F | CAGCAGCAGCCCGACCT | 122bp |
|  | HOX12-2R | ACGCGTACTCACTCATG |  |
| *GA2OX3* | GA2OX3-1F | GGTTCTTCAAGGTCGTC | 159bp |
|  | GA2OX3-1R | CGCCATTGAACCCGATCC |  |
| *Ubiquitin*  (LOC_Os05g06770) | Ubi-F | ATCGACAACGTGAAGGC | 195bp |
|  | Ubi-R | CTTGGTGTACGTCTTCTTCT |  |
